# Examiner perceptions of the MRCGP recorded consultation assessment for general practice licensing during COVID-19: cross-sectional study

**DOI:** 10.1186/s12909-023-04027-4

**Published:** 2023-01-26

**Authors:** Vanessa Botan, Despina Laparidou, Viet-Hai Phung, Peter Cheung, Adrian Freeman, Richard Wakeford, Meiling Denney, Graham R. Law, Aloysius Niroshan Siriwardena

**Affiliations:** 1 Community and Health Research Unit, School of Health and Social Care, University of Lincoln, Lincoln, LN5 7AT England; 2 Royal College of General Practitioners, 30 Euston Square, London, NW1 2FB UK; 3 University of Exeter Medical School, Exeter, EX1 2HZ England; 4 Hughes Hall, University of Cambridge, Cambridge, CB1 2EW England

**Keywords:** Education, medical, postgraduate, Assessment, Licensing, General practice, COVID-19

## Abstract

**Background:**

The Recorded Consultation Assessment (RCA) was developed rapidly during the COVID-19 pandemic to replace the Clinical Skills Assessment (CSA) for UK general practice licensing. Our aim was to evaluate examiner perceptions of the RCA.

**Methods:**

We employed a cross-sectional design using a questionnaire survey of RCA examiners with attitudinal (relating to examiners thoughts and perceptions of the RCA) and free text response options. We conducted statistical descriptive and factor analysis of quantitative data with qualitative thematic analysis of free text responses.

**Results:**

Overall, 182 of 260 (70%) examiners completed the questionnaire. Responders felt that consultations submitted were representative of the work of a typical GP during the pandemic and provided a good sample across the curriculum. They were also generally positive about the logistic, advisory and other support provided as well as the digital platform. Despite responders generally agreeing there was sufficient information available in video or audio consultations to judge candidates’ data gathering, clinical management, and interpersonal skills, they were less confident about their ability to make judgments of candidates’ performance compared with the CSA. The qualitative analysis of free text responses detailed the problems of case selection and content, explained examiners’ difficulties when making judgments, and detailed the generally positive views about support, training and information technology. Responders also provided helpful recommendations for improving the assessment.

**Conclusion:**

The RCA was considered by examiners to be feasible and broadly acceptable, although they experienced challenges from candidate case selection, case content and judgments leading to suggested areas for improvement.

**Supplementary Information:**

The online version contains supplementary material available at 10.1186/s12909-023-04027-4.

## Introduction

During the pandemic examination bodies have had to review exam processes to reduce the risk of COVID-19 infection to candidates and others involved, for example, in the case of medical objective structured clinical exams (OSCEs), the risks to examiners and patients or simulators [1]. In the United States (US) this led to the US Medical Licensing Examination (USMLE) being postponed [2], whereas other exams have been modified using socially distanced [3] or remote assessments [4, 5].

The Clinical Skills Assessment (CSA) is an OSCE undertaken at a test centre using simulators (actors), which together with the computer-based Applied Knowledge Test (AKT) and Workplace Based Assessment (WPBA), has formed the regulatory approved UK licensing exam for general practice, the Membership of the Royal College of General Practitioners (MRCGP), since 2008. The CSA, which includes 13 stations, each marked independently by two examiners, tested ‘a doctor’s ability to gather information and apply learned understanding of disease processes and person-centred care appropriately in a standardised context, make evidence-based decisions, and communicate effectively with patients and colleagues’ as well as also examining ‘candidates’ ability to integrate these skills effectively’ [6].

In the UK, many trainees were due to take the Clinical Skills Assessment (CSA) in April/May 2020 to complete training and to receive a licence to practice, and enter the qualified GP workforce by August 2020. With the temporary abandonment of the CSA because of the pandemic in March 2020, doctors would have been prevented from qualifying as general practitioners (GPs) without the development of a safe alternative.

Because of the risks involved in conducting a face-to-face OSCE, the CSA was replaced by the Recorded Consultation Assessment (RCA), developed in May 2020 by a specially convened team of educational, technical, assessment and psychometric experts and designed to assess the ability of GP specialist trainees to integrate and apply clinical, professional, communication and practical skills for general practice [7]. Before the introduction of the CSA the MRCGP used a video consultation assessment in which a videotape, consisting of seven consultations considered by candidates to best represent their ability to demonstrate 15 performance criteria, had each consultation assessed independently by seven different trained raters [8]. Video assessment has been previously used and validated for assessment of consultations in medical students [9] and doctors [10].

Apart from the use of a recorded consultation of real patients, the RCA differed considerably from the old MRCGP video assessment. The RCA uses real patient consultations conducted in the candidate’s working environment, with patient consent, during the pandemic. Candidates were able to choose, for each consultation, video (online or face-to-face contact) or audio (telephone contact) recordings. Thirteen consultations were required to align with the CSA which includes 13 face-to-face encounters, sometimes including one telephone consultation as an alternative. Each consultation was double marked providing 26 separate evaluations by different examiners for each candidate recording [11]. An initial technical pilot of the new assessment process and information technology platform (phase 1 involving 13 candidates) led to a further (phase 2) pilot with 1551 actual candidates between July and August 2020.

We sought to evaluate this major change to the assessment by assessing exam performance, psychometrics, and stakeholder experiences of candidates [11] and examiners. This study forms part of this evaluation and aimed to evaluate examiner experiences and perceptions of the phase 2 RCA pilot.

## Methods

### Research question and aim

Research question: What were the experiences and perceptions of examiners when conducting the RCA?

Aim: To explore the experiences and perceptions of examiners of the RCA .

### Design

We used a cross-sectional survey employing a purpose designed online questionnaire with quantitative and free text responses. We used the questionnaire variant of the convergent mixed methods design to gather and analyse both quantitative and free text (qualitative) data from the survey [12]. Ethical approval was sought and received by the Research Ethics Committee of the University of Lincoln School of Health and Social Care.

### Theory

We used a postpositivist stance in line with the mixed methods design, using critical realist theory which adopts the perspective that observations are imperfectly and probabilistically understood in light of factors such as experience, culture and social norms [13]. The theory is in line with the mixed methods survey design used to gather and analyse both quantitative and free text (qualitative) data [12].

### Setting

The RCA is a summative assessment of a doctor’s ability to integrate and apply clinical, professional, communication and practical skills appropriate for general practice, using pre-recorded video or audio consultations to provide evidence from a range of encounters in general practice relevant to the curriculum. Guidance at the time encouraged candidates to demonstrate their skills across a breadth of the curriculum by including a wide range of different consultations: no more than two cases from any one curriculum area, reflecting the frequency of presentation within general practice and the great variety of work GPs face; a spread of ages (including where possible a child and an older patient); a case involving a mental health concern; a case with a long-term condition e.g. cancer, multimorbidity or disability; and, a case related to urgent or unscheduled care. Candidates were encouraged to record new patient contacts rather than follow up patients as these were considered more likely to allow them to demonstrate competence in consultation skills. Each candidate recorded or uploaded 13 patient consultations onto a customised online information technology platform (FourteenFish: https://www.fourteenfish.com/partners/rcgp) for viewing and assessment by examiners. Examiners were provided with annual training and information detailing the exam policy, marking schedules, grade descriptors, the form in which to provide feedback to candidates, a summary of guidance and a marking aide-memoire [14].

### RCA examiners

Potential examiners applied to the panel following an advertisement on the RCGP website, by submitting an application supported by two referees who could attest to their clinical competence and professionalism. Following shortlisting against specific competencies, potential examiners were invited to attend a selection day, where they were assessed by a selection panel. Successful candidates undertook training. Mandatory criteria for selection included being in active clinical practice with no restrictions on practice from the regulatory body (the General Medical Council), a minimum of 5 years (whole-time equivalent) post-qualification experience as a GP, Membership or Fellowship of the RCGP and annual GP appraisal that met the requirements for medical revalidation. There were 250 examiners at the time of the study located across the UK including England (179, 71.6%), Scotland (19, 7.6%), Wales (15, 6.0%) and Northern Ireland (7.2.8%), with missing data for 30 (12%)%). Experience as an MRCGP examiner ranged from 3 to 16 years.

### Questionnaire construction

An online questionnaire survey (Table 1), consisting of a mix of positively and negatively framed experience and attitudinal items was specifically developed for the evaluation by members of the team (PC, AF, MD, AS) experienced in survey design. Responses were scored on a scale from 0 – strongly disagree to 4 - strongly agree. Questions asked examiners their views on: whether there was sufficient information in video and audio consultations to make a judgement of candidates’ data gathering, clinical management and interpersonal skills, whether consultations reflected the variety of GP work across the curriculum; the content of the consultations sampling across the curriculum; confidence in the judgements made compared to examining in the CSA; the online platform, standard setting and organisation. Free text options asked, Do you have any comments on the training? Do you have any comments on the marking? and Do you have any comments on the logistics (structure of the day, timetable, meetings, marshal support, IT)?

**Table 1:** Examiner questionnaire

What data are we collecting? The results from this survey will be used to evaluate the Recorded Consultation Assessment (RCA) only. This evaluation will be conducted by the RCGP. This survey will not be used to evaluate or assess you as an examiner. This survey is designed to be anonymous and as such, we ask that you do not provide any personal data or identifying information in any of the ‘free text’ answers. The data you provide will only be used for the purpose outlined above and stored by the RCGP (https://www.rcgp.org.uk/terms-and-conditions/privacy-statement.aspx) in compliance with all relevant Data Protection laws. This survey is designed with, and hosted by, Online surveys (run by Jisc). Online surveys is fully compliant with all UK data protection laws and details on the way they store and process the data you provide can be found here: https://www.onlinesurveys.ac.uk/terms-and-conditions/ Survey In the survey below you will see a series of statements. Please indicate the extent to which you agree or disagree with each statement. You will have the opportunity to provide free text comments at the bottom of this page. 1. For the video consultations that I viewed, there was generally sufficient information available for me to make a judgement of the candidates’ data gathering. Strongly agree : Agree : Neutral (neither agree nor disagree) : Disagree : Strongly disagree 2. For the video consultations that I viewed, there was generally sufficient information available for me to make a judgement of the candidates’ clinical management. Strongly agree : Agree : Neutral (neither agree nor disagree) : Disagree : Strongly disagree 3. For the video consultations that I viewed, there was generally sufficient information available for me to make a judgement of the candidates’ interpersonal skills. Strongly agree : Agree : Neutral (neither agree nor disagree) : Disagree : Strongly disagree 4. Did you mark any audio only consultations (i.e. consultations with no video)? Yes : No 4a. For the audio consultations that I viewed, there was generally sufficient information available for me to make a judgement of the candidates’ data gathering. Strongly agree : Agree : Neutral (neither agree nor disagree) : Disagree : Strongly disagree 4b. For the audio consultations that I viewed, there was generally sufficient information available for me to make a judgement of the candidates’ clinical management. Strongly agree : Agree : Neutral (neither agree nor disagree) : Disagree : Strongly disagree 4c. For the audio consultations that I viewed, there was generally sufficient information available for me to make a judgement of the candidates’ interpersonal skills. Strongly agree : Agree : Neutral (neither agree nor disagree) : Disagree : Strongly disagree 5. The content of the consultations I viewed was not reflective of the variety of a GP’s work during the Covid-19 pandemic. Strongly agree : Agree : Neutral (neither agree nor disagree) : Disagree : Strongly disagree 6. The content of the consultations I viewed represented a good sample across the curriculum. Strongly agree : Agree : Neutral (neither agree nor disagree) : Disagree : Strongly disagree 7. My level of confidence in the judgements I made compared to examining in the CSA is: Much better : Better : About the same : Worse : Much worse 8. It was difficult to use the 3-point borderline standard setting judgement. Strongly agree : Agree : Neutral (neither agree nor disagree) : Disagree : Strongly disagree 9. It was easy to use the 5-point standard setting system. Strongly agree : Agree : Neutral (neither agree nor disagree) : Disagree : Strongly disagree 10. The Hofstee standard setting questions were easy to answer. Strongly agree : Agree : Neutral (neither agree nor disagree) : Disagree : Strongly disagree 11. It was easy to view, assess and feedback using the FourteenFish platform. Strongly agree : Agree : Neutral (neither agree nor disagree) : Disagree : Strongly disagree 12. The morning briefing meeting was helpful. Strongly agree : Agree : Neutral (neither agree nor disagree) : Disagree : Strongly disagree 13. The RCA examiner training was helpful in preparing me for the examination. Strongly agree : Agree : Neutral (neither agree nor disagree) : Disagree : Strongly disagree 14. The marshal was accessible during the day. Strongly agree : Agree : Neutral (neither agree nor disagree) : Disagree : Strongly disagree 15. I felt supported by the marshal with I.T/recorded consultation issues. Strongly agree : Agree : Neutral (neither agree nor disagree) : Disagree : Strongly disagree 16. Queries/incidents were dealt with in a timely manner by the marshal. Strongly agree : Agree : Neutral (neither agree nor disagree) : Disagree : Strongly disagree 17. Reminders by the marshal for the submission of marks were a useful prompt. Strongly agree : Agree : Neutral (neither agree nor disagree) : Disagree : Strongly disagree *Free text* 18. Do you have any comments on the training? 19. Do you have any comments on the marking? 20. Do you have any comments on the logistics (structure of the day, timetable, meetings, marshal support, IT)?	

### Participants

The survey was offered to all RCA examiners who were invited by email in July and August 2020 to complete the questionnaire on a voluntary basis once they had assessed consultations. Written consent was sought and gained.

### Statistical analysis

Descriptive statistical tests of numbers and percentages were used to summarise responses given on a four-point scale ranging from strongly disagree to strongly agree (Table 2). These were employed to explore and describe examiners’ perceptions of the RCA for each item. Negatively framed items (Questions 5 and 8 in Table 1) were recoded so that higher scores indicated more positive attitudes.

**Table 2:** Examiner responses to questionnaire

Item Name	Strongly Disagree	Disagree	Neither Agree nor Disagree	Agree	Strongly Agree
1. For the video consultations that I viewed, there was generally sufficient information available for me to make a judgement of the candidates’ data gathering	1 0.55%	12 6.59%	16 8.79%	123 67.58%	30 16.48%
2. For the video consultations that I viewed, there was generally sufficient information available for me to make a judgement of the candidates’ clinical management	2 1.10%	9 4.95%	30 16.48%	118 64.84%	23 12.64%
3. For the video consultations that I viewed, there was generally sufficient information available for me to make a judgement of the candidates’ interpersonal skills	0	4 2.20%	8 4.40%	118 64.84%	52 28.57%
4.a. For the audio consultations that I viewed, there was generally sufficient information available for me to make a judgement of the candidates’ data gathering	1 0.57%	10 5.75%	15 8.62%	123 70.69%	25 14.37%
4.b. For the audio consultations that I viewed, there was generally sufficient information available for me to make a judgement of the candidates’ clinical management	2 1.15%	16 9.20%	32 18.39%	108 62.07%	16 9.20%
4.c. For the audio consultations that I viewed, there was generally sufficient information available for me to make a judgement of the candidates’ interpersonal skills	2 1.15%	16 9.20%	32 18.39%	108 62.07%	16 9.20%
5. The content of the consultations I viewed was not representative of the work of a typical GP during the COVID-19 pandemic^b^	32 17.58%	100 54.95%	31 17.03%	15 8.24%	4 2.20%
6. The content of the consultations I viewed represented a good sample across the curriculum	5 2.75%	52 28.57%	39 21.43%	78 42.86%	8 4.40%
7. My level of confidence in the judgements I made compared to examining in the CSA is:^a^	3 1.65%	66 36.26%	104 57.14%	8 4.40%	1 0.55%
8. It was difficult to use the 3-point borderline standard setting judgement^b^	23 12.57%	102 55.74%	36 19.67%	20 10.93%	2 1.09%
9. It was easy to use the 5-point standard setting system.	4 2.19%	29 15.85%	32 17.49%	94 51.37%	24 13.11%
10. The Hofstee standard setting questions were easy to answer.	27 14.84%	54 29.67%	58 31.87%	37 20.33%	6 3.30%
11. It was easy to view, assess and feedback using the FourteenFish platform.	1 0.55%	4 2.20%	4 2.20%	55 30.22%	118 64.84%
12. The morning briefing meeting was helpful.	2 1.10%	11 6.04%	28 15.38%	108 59.34%	33 18.13%
13. The RCA examiner training was helpful in preparing me for the examination.	0	4 2.20%	8 4.40%	81 44.51%	89 48.90%
14. The marshal was accessible during the day.	0	2 1.09%	15 8.20%	67 36.61%	99 54.10%
15. I felt supported by the marshal with I.T/recorded consultation issues.	1 0.55%	1 0.55%	43 23.63%	63 34.62%	74 40.66%
16. Queries/incidents were dealt with in a timely manner by the marshal.	0	3 1.65%	37 20.33%	56 30.77%	86 47.25%
17. Reminders by the marshal for the submission of marks were a useful prompt.	2 1.09%	13 7.10%	106 57.92%	45 24.59%	17 9.29%

^a^the scale for Q7 is: much worse, worse, same, better, much better

^b^items 5 and 8 were reverse coded. Q4d Did you mark any audio only consultations (i.e. consultations with no video)? No: 8 (4.4%); Yes:174 (95.60%)

Because of the multitude and variety of items, an exploratory factor analysis was conducted to reduce the number of items and to uncover possible underlying factors or subscales of the questionnaire. As such, this analysis identified highly correlated items and grouped them into subscales. Cronbach’s alpha was then calculated for each subscale to check its internal consistency. The exploratory factor analysis using varimax rotation was conducted after checking if factor analysis was suitable as indicated by Kaiser-Meyer-Olkin (KMO) values higher than 0.6 and retaining factors with eigenvalues higher than 1 [15]. Cronbach’s alpha was calculated for identified factors (subscales).

**Fig. 1:**
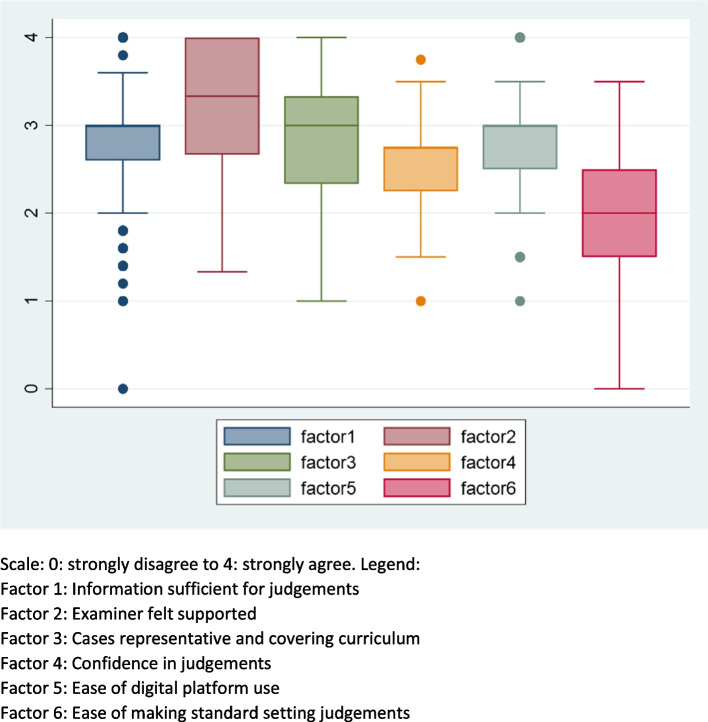
Median and interquartile range and outlier scores for factors. Scale: 0: strongly disagree to 4: strongly agree. Legend: Factor 1: Information sufficient for judgements. Factor 2: Examiner felt supported. Factor 3: Cases representative and covering curriculum. Factor 4: Confidence in judgements. Factor 5: Ease of digital platform use. Factor 6: Ease of making standard setting judgements

Box plots were used to show medians, spread and skewness of the data and its variability as indicated by interquartile ranges (IQR) for each factor (Fig. 1).

The rate of missing data was very low (there were only eight missing data points for question 4a, b, and c) and these were excluded from the analysis. There were no extreme outliers and as such all responses were kept because they represented viable answers to all questions within the range of strongly disagree to strongly agree. Data were analysed using the statistical software Stata 15.1.

The initial sample size calculated for the study after setting alpha at 0.05, power at 0.8 and a medium effect size of 0.3 was 64 [16].

### Qualitative analysis of free text responses

Thematic analysis supported by NVivo 12 was undertaken for free text responses. Responses were coded and organised into themes by three experienced qualitative researchers (DL, VP, NS) using a multistage approach: (1) line by line coding of free text responses independently by DL and VP (2) organisation of codes into categories through discussion (DL, VP, AS), and (3) grouping of categories into themes after discussion (AS, supported by DL and VP).

## Results

### Responders

There were responses from 182 of 260 (70%) examiners sent the questionnaire. Demographic characteristics of participants were not collected to maintain anonymity and because it had little relevance for the analysis.

### Quantitative results

Most responders felt there was enough information available to make a judgement of candidates’ data gathering, clinical management, and interpersonal skills for both video and audio consultations. The percentage of examiners that agreed or strongly agreed that they had enough information was over 60% for all items exploring this aspect (questions 1 to 4c in Table 2). They mostly agreed consultations were representative of the work of a typical GP during the COVID-19 pandemic and sampled well across the curriculum as indicated by the responses to questions 5 and 6 in Table 2. They were generally positive about the logistic, advisory and other support provided and the digital platform. They were more negative about their confidence in making judgments compared to the CSA and in standard setting with less than 5% considering that their judgement was better, 57% that it was the same and over 38% that it was worse (Table 2).

Exploratory factor analysis identified six factors or subscales (Table 3). These subscales allowed us to better group the responses and draw more general conclusions about examiners’ perceptions, and half of them had good internal consistency (Table 4).

**Table 3:** Questionnaire subscales: factor analysis

Item	Factor1	Factor2	Factor3	Factor4	Factor5	Factor6
Q4c	**0.9272**	0.0135	0.0772	0.0236	−0.0818	− 0.0669
Q4b	**0.9272**	0.0135	0.0772	0.0236	− 0.0818	− 0.0669
Q2	**0.8309**	0.0865	0.1271	0.0847	0.1086	− 0.0222
Q1	**0.7047**	− 0.0106	0.0883	− 0.0435	0.1015	0.3578
Q4a	**0.6825**	− 0.0887	0.2405	− 0.0234	0.1815	0.2670
Q15	0.0215	**0.8430**	0.1206	− 0.0594	− 0.0168	0.1320
Q14	−0.0035	**0.8359**	− 0.0195	0.0267	0.0806	− 0.0641
Q16	−0.0087	**0.8351**	0.1045	0.1407	0.0962	0.0037
Q6	0.2913	0.1060	**0.7193**	0.1931	0.0932	0.0625
Q5	−0.2210	−0.0607	**−0.7101**	0.0723	0.0713	0.0301
Q13	0.1091	0.2415	**0.5076**	−0.3921	−0.0320	0.1899
Q9	0.0695	0.1869	0.0493	**0.5888**	−0.2285	0.0261
Q7	0.2862	0.1151	0.2705	**0.4775**	− 0.2576	0.0942
Q12	0.1569	0.3746	0.2648	**− 0.4393**	− 0.3106	− 0.0062
Q3	0.3768	0.2218	0.0375	**0.3771**	0.2441	0.2422
Q11	0.0767	0.2421	− 0.0319	−0.1202	**0.7787**	0.0503
Q17	0.1302	0.4363	−0.2597	− 0.1682	**− 0.4928**	0.2253
Q8	− 0.0616	− 0.2483	− 0.1240	−0.1744	0.0423	**− 0.6960**
Q10	0.1618	0.3103	0.1020	0.3075	− 0.0535	**− 0.5556**

**Table 4:** Internal consistency (Cronbach’s α) for factors

All_items	0.79
**Factor 1: Information sufficient for judgements**	0.90
**Factor 2: Examiner_felt_supported**	0.85
**Factor 3: Cases representative and covering curriculum**	0.54
**Factor 4: Confidence in judgements**	0.32
**Factor 5: Ease of digital platform use**	0.06
**Factor 6: Ease of making standard setting judgements**	0.08

Remarks:

All items, except Q3 had adequate factor loadings above 0.4

Factor 1 and 2 had high internal consistency, with α ≥ 0.8^a^

Factor 3 had lower consistency but may be considered acceptable for an exploratory study

Factors 4,5, and 6 had very low consistency and should be interpreted with caution

^a^NB an α ≥ 0.8 is considered acceptable; an α ≥ 0.7 is considered acceptable for an exploratory study

The overall internal consistency of the questionnaire was good (α = 0.79). The internal consistency of subscales 1 and 2 was also very good (α > 0.8) and of the third subscale was acceptable (α = 0.54) The internal consistency of factors 4,5, and 6 was very low indicating a lower level of correlation between items in these factors (Table 4).

There were three main subscales identified which summarised how sufficient the information was for making a judgement (Factor 1), how supported the examiners felt (Factor 2), and how well cases covered the curriculum (Factor 3). The distribution of the data for the first subscale, as shown in Fig. 1, indicated that examiners agreed that the information was sufficient to make a judgement. The median of this subscale was three and there were only six outliers that scored below 2, meaning that they disagreed with the fact that the information was sufficient. Factor 2 corresponding to the support that the examiners received in the process of marking the RCA had the highest mean indicating that examiners felt highly supported and that the training and resources were helpful. The examiners also considered that the consultations covered well the curriculum with most of them agreeing that this was true as indicated by the density of scores above 2 (Fig. 1). The examiners also tended to agree with the fact that it was easy to use the digital platform as shown by Factor 5 in Fig. 1.

Factor 6 ‘Ease of making standard setting judgements’ had the lowest median of, with most scores gravitating below it indicating that examiners tended to disagree with the statement that it was easy to make standard setting judgements and that they were more equivocal in their confidence about the standard setting judgments.

### Free text themes

The questions 18. Do you have any comments on the training? 19. Do you have any comments on the marking? and 20. Do you have any comments on the logistics (structure of the day, timetable, meetings, marshal support, IT) elicited 121 (18),137 (Q19) and 141 (Q20) comments from respondents (denoted examiner E1–183 in the example quotes). Four themes were identified: 1. Problems with case selection and content; 2. Varying ease of making judgments; 3. Generally positive views of support, training and information technology support; and 4. Recommendations for cases, candidates, judgments and support. (see supplementary Table S1).

#### Theme 1: problems with case selection and content

Problems of variation in cases selected and put forward for assessment and limitations in case content, partly explained by the lack of time candidates had to collect and submit cases, were a barrier to assessment.

##### Variability in case selection

Unsuitable cases: ‘Worried about self-selection of cases as being quite different from cases as in CSA with the guaranteed spread of the curriculum.’ E98. ‘Choice of cases was variable - some clearly unsuitable (e.g. pill reviews, referral requests, no diagnosis to be made etc.).’ E140.

Variable consultation complexity: ‘Much harder than with the CSA to be fair between candidates due to the differing complexity of the consultations.’ E36.

Lack of standardised challenge: ‘The big issue is the case content; they do reflect experience of practice; but not really the curriculum as per CSA; plus lack of standardised challenge.’ E64.

##### Limited case content

Lack of information: ‘Unless the examiner has the knowledge of what has been seen or heard in consultation, it is quite difficult to assess the clinical management.’ E145. ‘Often needed more info e.g. photos submitted or what blood tests were.’ E61.

Lack of evidence: ‘Descriptors don’t allow for lack of demonstration, hence more difficult to fail for lack of evidence.’ E99. ‘It was not always easy to judge the correctness of any diagnoses or plans as you did not have any background information or the findings other than what the trainee vocalised. Some patients had problems which needed further assessment, and while this was appropriate it didn’t make it easy to assess their management.’ E111.

Lack of domain coverage: ‘Lack of consultations covering the domains frequent.’ E182. ‘It was difficult to mark the data gathering on the videos because the candidate had usually seen the patient first and got a lot of the hx [history] first. The telephone consultations were difficult to assess management because the candidate would often just ask the patient TCI [to come in] without explaining what they were going to do.’ E94.

Low challenge: ‘Some candidates made a mountain out of the simplest of cases and appeared to baffle the patients.’ E181. ‘Candidates should not be rewarded for turning a 2-minute consult into 10.’ E108.

##### Limited time to collect and submit cases

Lack of time for candidates: given the pandemic and the difficulty in finding suitable cases in such a short time frame. E111.

#### Theme 2: varying ease of making judgments

##### Compared with CSA

Some examiners found the RCA easier to assess compared to the CSA whereas others found it similar or more difficult. The CSA was felt by some to allow more complex cases to be assessed but other found some aspects, such a clinical management, were better assessed in the RCA.

Easier: ‘Day felt much more relaxed than a CSA marking day. I could mark at home and work at my speed. Easy to mark consultations apart from low challenge cases which need some guidance.’ E40.

Similar: ‘Pleased it did not differ from marking the CSA which I’m experienced in.’ E139. ‘Marking was similar to the CSA and hence I knew what I was looking for.’ E44.

More difficult: ‘Due to the cases presented the CSA marking schedule for Clinical management often did not apply. The CSA cases are designed to examine this aspect much better than the RCA in my opinion.’ E84.

Allows more complex cases to be assessed: RCA allows assessment of multiple morbidity/ large agenda cases which are difficult to write for the CSA.’ E149.

##### Assessment criteria and marking

There were many comments that marking cases was straightforward. Double marking and multiple judgments were welcomed to ensure concordance and fairness, but some were concerned by the lack of calibration to ensure standardisation of marking and domains that were perceived to be more difficult to assess.

Straightforward: ‘Straightforward having completed the training. Not sure why a few examiners seemed to have trouble with e.g. low challenge as we had clear guidance.’ E141.

Double marking welcomed: ‘I’m glad there was double marking and after sensible threshold (e.g. 200 cases) it would be helpful to have some feedback on our concordance.’ E146.

Lack of confidence balanced by multiple judgments: ‘I am quite insecure about the validity and consistency of my marks but take reassurance that each trainee is assessed by 26 examiners.’ E177.

Standardisation: The lack of standardisation and calibration made me feel uneasy in what is a very high stakes exam. E35.

Some assessment domains appeared more difficult: ‘Management was often hard to assess as candidates often just arranged for patient to be seen. Long explanations of how to gain access to the building but often very little on what they might do, why and what would happen next.’ E60.

##### Effect of case selection on judgments

Case selection was felt to influence marking. Responders varied in finding some, particularly low challenge or complex, cases and consultations which were unbalanced in their content (clinical management vs data gathering) more difficult to assess or to spread grades.

Case selection affected marking: ‘Marking was influenced by the choice of consultations submitted. Some consultations were easier to mark across all 3 domains. Low challenge consultations, reviewing a colleague’s previously implemented treatment or previously triaged consultations made marking DG [data gathering] in particular (and also CM [Clinical Management]) more difficult.’ E104.

Low challenge and complex cases more difficult to assess: ‘Apart from the low challenge cases that have been often discussed - there also is a challenge to mark more complex cases where there are 2 or 3 things running and they are trying to complete them in the 10 mins - do some but not others - at times the more important one is not concluded.’ E25.

More complex cases easier to assess: Some candidates made a mountain out of the simplest of cases and appeared to baffle the patients. My overall thoughts were more complex cases were easier to mark and that patients are very tolerant.’ E181.

Difficult to mark already known problems or patient self-diagnosis: ‘Find that candidates presenting cases that are known problems or when a patient has self-diagnosed and they are seeking confirmation of diagnosis very difficult to award marks in data gathering and IPS [interpersonal skills].’ E76.

Spread of grades more difficult: ‘Case selection meant that it was difficult at times to spread the marks, with quite a lot of consultations running down the middle grades.’ E180.

Balance of content affected decision: ‘Difficulty with marking ‘unbalanced cases’ i.e. lots of data gathering needed but little management - or the reverse- as this does not happen in the CSA.’ E144.

##### Consultation format and timing

The format, particularly telephone consultations, and the restriction to 10 minutes limited the content and thus the assessment that could be made.

Audio difficult: “Difficulty with marking management on audio calls”. E144.

Lack of data on telephone calls: “Telephone consultations quite often one outcome was to organise f2f consultation later in the day. This therefore missed out management and diagnosis, although some candidates stated what they might do”. E118.

Lack of content in short consultations: ‘I don’t think the 10-min rule was fair on the candidates. Those candidates who kept to 10 min in my experience had submitted recordings of cases that could be considered low challenge or somewhat “staged” e.g. a simple pill check or review of a well-controlled condition.’ E166.

#### Theme 3: generally positive views of support, training and information technology support

There were overall positive views of support, training and information technology with generally minor problems.

##### Generally positive views

Well organised despite short development time: ‘Really impressed you managed to get this up and running without any IT issues on the days I examined.’ E112.

Concerns about retention: ‘I think examiner retention may be an issue if remote working were to continue post Covid-19.’ E12.

Effective online system and information technology support: ‘The IT and general support were excellent.’ E113. IT/FourteenFish really impressive. E135.

##### Training

The many positive comments about training were tempered with the need for ongoing support to deal with specific problems which arose for examiners.

Positive about training sometimes with qualification: ‘Excellent training module and practice cases.’ E112. ‘It was well-designed, in so far as an online resource allows. However, my general observation is that I was not fully prepared for the RCA prior to hitting the floor and that most learning took place as a result of being actively involved in marking.’ E180.

Specific problems required advice: ‘The training was useful for giving an overview, but quite general whereas the problems encountered tended to be quite specific.’ E111. ‘The training was helpful. However, a myriad of other queries came up once actual cases were started.’ E144.

Good support and response: ‘It all ran smoothly. I was worried if I asked the marshal anything I would be unable to progress to the next case until I had an answer. In fact I got fast replies so not an issue.’ E60.

##### Meetings

Meetings were generally felt to be helpful, particularly when well-led but some meetings were felt to be too long, unnecessary or lacking purpose, and sometimes groups were perceived to be too large with little opportunity for engagement.

Meetings helpful when effectively led: ‘Some of meetings less useful than others: very dependent on leadership.’ E164 ‘The lunchtime and 5pm Zoom meetings worked best when effectively chaired to stop examiners going over ground already covered in the morning briefing and in training.’ E131.

##### Time for marking sufficient vs tight

Examiners varied in whether they thought there was sufficient time for marking.

Sufficient time: ‘In general, I would say the day worked very well and there was sufficient time to mark all 26 candidates.’ E22.

Tight timescales: Marking the cases was sometimes a bit tight; I had to skip mornings breaks to get ahead in order to complete the afternoon marking; so shorter briefing would help. E10.

#### Theme 4: recommendations for cases, candidates, judgments and support

Several recommendations were put forward to remedy any shortfalls seen in the assessment.

##### Recommendations for case selection and candidate information

Advice on case selection: ‘Some areas such as type of consultations submitted, and range of cases need refining.’ E170.

Clearer information for candidates: **‘**The other issue is about cheating or ‘That is what we do in normal practice ‘- some candidates were looking at information while doing the consultation. If it is acceptable, then every candidate should be informed of that and there should be equality (the fairness we promote all the time).’ E145.

##### Recommendations for examiner judgments

More information provided on cases: ‘I would have liked candidates to be able to submit the photos of lesions etc. to enable me to assess management better.’ E8. ‘It would be helpful to have more information in the workbook in some of the cases re PMH [past medical history] of patient.’ E55.

More calibration and benchmarking: ‘More calibration clips would have been really helpful. We did not find out how our own marks compared against the expert panel. More clips akin to the palette we saw (e.g. low challenge cases) would have helped us to feel more reliable and consistent across examiners in our judgements on the day.’ E42. ‘Needs more training audio recordings/videos with agreed scores and justifications as a way to set some sort of benchmark.’ E21.

Clearer pass/fail descriptors: ‘The descriptors for the different domains would benefit from more clearly showing that the candidate needs to do enough to demonstrate competence i.e. that doing all that is needed in a low challenge case is not sufficient to gain a pass. The feedback statements need to reflect the above.’ E100 ‘Many consultations were a demonstration of consultation skills and not of clinical decision making. Often history taking was ‘tell me more’ and ‘any other symptoms’ and very formulaic with little evidence of focused history taking. Often there was a pre-existing diagnosis which the candidate simply confirmed. I think we are all agreed that we need new ‘word pictures’ to describe passing/failing.’ E86.

Modify scoring to reflect consultation difficulty: ‘Change the generic descriptors to clarify that marks must be gained with the candidate starting with zero and gaining marks as they show skills. Telephone triage consults should score poorly because they can’t lead to a definitive management plan. Giving the treatment the patient asked for should score zero in CM [Clinical Management] if no additional value is added during the consultation. Candidates should not be rewarded for turning a 2-minute consult into 10.’ E108.

Better feedback to examiners: ‘It would have been nice to get some feedback on the calibration exercise and some discussion on Zoom with fellow examiners would have been useful as part of the training.’ E33.

Improved feedback to candidates: ‘We need some better feedback statements e.g. insufficient challenge to demonstrate skills, insufficient new management demonstrated. Some of the statements should also be in data collection e.g. insufficient psychosocial information to put problem in context.’ E102.

Enhance training: ‘Training was good but the RCA needs to change really, and hence examiners will need further training.’ E177. ‘It would be good to have had examples of low challenge cases as these were the hardest to assess. It would be nice to know how other people marked the cases to give a bit of bench marking, and even some discussion of why they marked that way.’ E151.

##### Recommendations for training and support

Alter balance of morning and afternoon cases: ‘Good to do a few afternoon cases in the morning.’ E76.

Reduce meetings: ‘I have been very impressed by his well it has worked. I think now the meetings are becoming less useful as we become more familiar with the processes.’ E77.

More one-to-one support: ‘Some of the issues raised would have been better dealt with on a one to one basis.’ E78.

## Discussion

### Main findings

We describe the experiences and perceptions of examiners when conducting the RCA, using a questionnaire survey. Examiners who responded to the questionnaire largely felt that consultations submitted by candidates were representative of the work of a typical GP during the pandemic and provided a good sample across the curriculum. They were also generally positive about the logistic, advisory and other support provided as well as the digital platform. Despite responders generally agreeing there was enough information available in video or audio consultations to assess candidates’ data gathering, clinical management, and interpersonal skills, they were less confident about their ability to make judgments of candidates’ performance. This was not surprising given that this was a newly developed licensing exam using non-standardised cases. The qualitative analysis of free text responses enabled an exploration of quantitative findings, detailing the problems found with case selection and content, explaining the difficulties in making judgments, and expanding on the generally positive views of support, training and information technology. Responders also provided helpful recommendations for improving the assessment.

### Strengths and limitations

The key strengths of this study were a good response rate, the questionnaire showing good reliability overall, and the triangulation of quantitative and qualitative findings. We did not collect demographic data from responders to the survey because of a risk, albeit small, that this would lead to possible identification and therefore reduce the response rate. We were therefore unable to explore the influence of examiner characteristics on experiences and perceptions.

### Comparison with existing literature

COVID-19 has affected the conduct of high stakes medical assessments worldwide leading to disruptive innovation to adapt exams to the current situation [1, 17]. The alternative, as adopted in the US, of suspending the United States Medical Licensing Examinations has led to mental distress and burnout among trainees [18]. The RCA was designed, as a temporary measure, to replace the CSA as a way of ensuring that doctors could continue to be licensed and qualify as GPs in the face of the infection risk to candidates, examiners and (simulated) patients during the pandemic. The video assessment was used in preference to socially distanced [3] or remote (tele-) OSCEs which have been successfully used elsewhere and have been found to perform as well as standard OSCEs [5].

The RCA was selected from a list of options given to stakeholders, which took into account feasibility, cost, and the ability to cater for candidates who were shielding or working from home. The assessment format for the RCA also benefitted from the UK experience of the previous video exam that preceded the CSA [19]. The RCA incorporated elements of the previous MRCGP video assessment that were found to contribute to that assessment’s validity [8], while increasing case numbers from 7 in the previous video exam to 13 in the RCA to enhance reliability and precision.

In a recent study of candidates’ taking the RCA, survey responders were also generally positive about the digital platform despite technical teething problems and were also positive about resources and support provided although contradictory or late guidance, insufficient time to gather cases, and logistic, equipment and cost barriers were noted [11]. A small majority of respondents felt the RCA was a fair assessment of clinical skills, but their experiences of the RCA were less positive, with a larger majority, particularly affecting those in smaller practices with socioeconomic deprivation or language barriers, expressing difficulty accessing and submitting cases and negative impacts on trainees, training, work, and patients described [20]. Candidate felt that the assessment would be improved by increasing the time to record and submit cases, greater time per case, allowing supporting information and better guidance, support, feedback and suggested changes to the online platform [11, 20].

### Implications for policy, practice and research

This study provides evidence that the RCA is a feasible alternative to the CSA during the COVID-19 pandemic, but also shows areas for improvement from an examiner perspective. Some of these suggestions have already been implemented, partly as a result of these findings. For example, the length of time allowed for each case has been increased from 10 minutes to 12 minutes [7], and the lack of time to record and submit cases due to the rapidly developed assessment, has increased in later exams. Improved guidance on case selection (for example, mandatory case criteria linked to clinical topic areas), and better support and feedback to examiners and candidates has been implemented [7].

.Future options for performance assessments for the MRCGP are currently under review and a detailed assessment of differential attainment in the RCA, which has been submitted to the UK regulator (the General Medical Council), will be published separately (personal communication).

Further research is needed to evaluate from the perspective of all stakeholders, the validity, reliability and precision of the RCA as it is developed and improved.

## Conclusions

Examiners felt that consultations for the RCA reflected GP work and the curriculum, were positive about the digital platform but were less confident about their judgments of candidates’ performance. The RCA was a feasible and broadly acceptable alternative to the CSA but had shortcomings perceived by examiners and showed areas for potential improvement and further evaluation.

## Supplementary Information

**Additional file 1: Table S1.** Themes and codes.

## Data Availability

The data that support the findings of this study are available from the RCGP, but restrictions apply to the availability of these data, which were used under license for the current study, and so are not publicly available. Data are however available from the authors upon reasonable request and with permission of the RCGP.
