# Supplementary material for: Examiner perceptions of the MRCGP recorded consultation assessment for general practice licensing during COVID-19: cross-sectional study
Source: BMC Med Educ. 2023 Jan 26;23:65. doi: 10.1186/s12909-023-04027-4 (PMC9879559; doi:10.1186/s12909-023-04027-4)
Supplement: Supplementary file 1 — Additional file 1: Table S1. Themes and codes. [file 12909_2023_4027_MOESM1_ESM.docx]

**Table S1 Themes and codes**

**Theme 1 Problems with case selection and content**

Problems of variation in cases selected and put forward for assessment and limitations in case content, partly explained by the lack of time candidates had to collect and submit cases, were a barrier to assessment.

*Variability in case selection*

Unsuitable cases: ‘Worried about self-selection of cases as being quite different from cases as in CSA with the guaranteed spread of the curriculum.’ E98. ‘Choice of cases was variable - some clearly unsuitable (e.g. pill reviews, referral requests, no diagnosis to be made etc.).’ E140.

Variable consultation complexity: ‘Much harder than with the CSA to be fair between candidates due to the differing complexity of the consultations.’ E36

Lack of standardised challenge: ‘The big issue is the case content; they do reflect experience of practice; but not really the curriculum as per CSA; plus lack of standardised challenge.’ E64.

*Limited case content*

Lack of information: ‘Unless the examiner has the knowledge of what has been seen or heard in consultation, it is quite difficult to assess the clinical management.’ E145. ‘Often needed more info e.g. photos submitted or what blood tests were.’ E61.

Lack of evidence: ‘Descriptors don't allow for lack of demonstration, hence more difficult to fail for lack of evidence.’ E99. ‘It was not always easy to judge the correctness of any diagnoses or plans as you did not have any background information or the findings other than what the trainee vocalised. Some patients had problems which needed further assessment, and while this was appropriate it didn't make it easy to assess their management.’ E111

Lack of domain coverage: ‘Lack of consultations covering the domains frequent.’ E182. ‘It was difficult to mark the data gathering on the videos because the candidate had usually seen the patient first and got a lot of the hx [history] first. The telephone consultations were difficult to make management because the candidate would often just ask the patient TCI [to come in] without explaining what they were going to do.’ E94

Low challenge: ‘Some candidates made a mountain out of the simplest of cases and appeared to baffle the patients.’ E181. ‘Candidates should not be rewarded for turning a 2 minute consult into 10.’ E108

*Limited time to collect and submit cases*

Lack of time for candidates: Overall it did not feel like a very fair assessment given the pandemic and the difficulty in finding suitable cases in such a short time frame. E111.

**Varying ease of making judgments**

*Compared with CSA*

Some examiners found the RCA easier to assess compared to the CSA whereas others found it similar or more difficult. The CSA was felt by some to allow more complex cases to be assessed but other found some aspects, such a clinical management, were better assessed in the CSA.

Easier: ‘Day felt much more relaxed than a CSA marking day. I could mark at home and work at my speed. Easy to mark consultations apart from low challenge cases which need some guidance.’ E40

Similar: ‘Pleased it did not differ from marking the CSA which I’m experienced in.’ E139. ‘Marking was similar to the CSA and hence I knew what I was looking for.’ E44

More difficult: ‘Due to the cases presented the CSA marking schedule for Clinical management often did not apply. The CSA cases are designed to examine this aspect much better than the RCA in my opinion.’ E84

Allows more complex cases to be assessed: RCA allows assessment of multiple morbidity/ large agenda cases which are difficult to write for the CSA.’ E149

*Assessment criteria and marking*

There were many comments that marking cases was straightforward. Double marking and multiple judgments were welcomed to ensure concordance and fairness, but some were concerned by the lack of calibration to ensure standardisation of marking. Some domains were perceived to be more difficult to assess.

Straightforward: ‘Straightforward having completed the training. Not sure why a few examiners seemed to have trouble with e.g. low challenge as we had clear guidance.’ E141

Double marking welcomed: ‘I'm glad there was double marking and after sensible threshold (e.g. 200 cases) it would be helpful to have some feedback on our concordance.’ E146

Lack of confidence balanced by multiple judgments: ‘I am quite insecure about the validity and consistency of my marks but take reassurance that each trainee is assessed by 26 examiners.’ E177

Standardisation: The lack of standardisation and calibration made me feel uneasy in what is a very stakes exam. E35

Some assessment domains more difficult: ‘Management was often hard to assess as candidates often just arranged for patient to be seen. Long explanations of how to gain access to the building but often very little on what they might do, why and what would happen next.’ E60

*Effect of case selection on judgments*

Case selection was felt to influenced marking. Responders varied in finding some, particularly low challenge or complex, cases and consultations which were unbalanced in their content (clinical management vs data gathering) more difficult to assess or to rank order.

Case selection affected marking: ‘Marking was influenced by the choice of consultations submitted. Some consultations were easier to mark across all 3 domains. Low challenge consultations, reviewing a colleague's previously implemented treatment or previously triaged consultations made marking DG [data gathering] in particular (and also CM [Clinical Management]) more difficult.’ E104

Low challenge and complex cases more difficult to assess: ‘Apart from the low challenge cases that have been often discussed - there also is a challenge to mark more complex cases where there are 2 or 3 things running and they are trying to complete them in the 10 mins - do some but not others - at times the more important one is not concluded.’ E25

More complex cases easier to assess: Some candidates made a mountain out of the simplest of cases and appeared to baffle the patients. I don’t know how a feedback statement would fit this though! My overall thoughts were more complex cases were easier to mark and that patients are very tolerant.’ E181

Difficult to mark already known problems or patient self-diagnosis: ‘Find that candidates presenting cases that are known problems or when a patient has self-diagnosed and they are seeking confirmation of diagnosis very difficult to award marks in data gathering and IPS [interpersonal skills].’ E76

Rank ordering more difficult: ‘Case selection meant that it was difficult at times to spread the marks, with quite a lot of consultations running down the middle grades.’ E180

Balance of content affected decision: ‘Difficulty with marking ‘unbalanced cases’ i.e. lots of data gathering needed but little management - or the reverse- as this does not happen in the CSA.’ E144

*Consultation format and timing*

The format, particularly telephone consultations, and the restriction to 10 minutes limited the content and thus the assessment that could be made.

Audio difficult: Difficulty with marking management on audio calls. E144

Lack of data on telephone calls: Telephone consultations quite often one outcome was to organise f2f consultation later in the day. This therefore missed out management and diagnosis, although some candidates stated what they might do. E118

Lack of content in short consultations: ‘I don't think the 10-min rule was fair on the candidates. Those candidates who kept to 10 min in my experience had submitted recordings of cases that could be considered low challenge or somewhat "staged" e.g. a simple pill check or review of a well-controlled condition.’ E166

**Generally positive views of support, training and information technology support**

There were overall positive views of support, training and information technology with generally minor problems.

*Generally positive views*

Well organised despite short development time: ‘Really impressed you managed to get this up and running without any it issues on the days I examined.‘ E112.

Concerns about retention: ‘I think examiner retention may be an issue if remote working were to continue post Covid-19.’ E12

Effective online system and information technology support: ‘The IT and general support were excellent.’ E113. IT/FourteenFish really impressive. E135

*Training*

The many positive comments about training were tempered with the need for ongoing support to deal with specific problems which arose for examiners.

Positive about training sometimes with qualification: ‘Excellent training module and practice cases.’ E112. ‘It was well-designed, in so far as an online resource allows. However, my general observation is that I was not fully prepared for the RCA prior to hitting the floor and that most learning took place as a result of being actively involved in marking.’ E180

Specific problems required advice: ‘The training was useful for giving an overview, but quite general whereas the problems encountered tended to be quite specific.’ E111. ‘The training was helpful. However, a myriad of other queries came up once actual cases were started.’ E144

Good support and response: ‘It all ran smoothly. I was worried if I asked the marshal anything I would be unable to progress to the next case until I had an answer. In fact I got fast replies so not an issue.’ E60

*Meetings*

Meetings were generally felt to be helpful, particularly when well-led but some meetings were felt to be too long, unnecessary or lacking purpose, and sometimes groups were perceived to be too large with little opportunity for engagement.

Meetings helpful when effectively led: ‘Some of meetings less useful than others: very dependent on leadership.’ E164 ‘The lunchtime and 5pm Zoom meetings worked best when effectively chaired to stop examiners going over ground already covered in the morning briefing and in training.’ E131.

Some meetings too long, unnecessary or lacked purpose: ‘Some of the zoom meetings were too long. Some of the issues raised would have been better dealt with on a one to one basis.’ E78 . ‘I am not sure that meetings were necessary at both 13.00 and 17.00.’ E113. ‘The purpose of the 1pm and 5pm sessions was not clear to me. Some were very brief while some ended up being a general philosophical chat about the exam.’ E103

Concerns not always listened to: ‘Felt that contributions at zoom meetings were either dismissed or ignored and that we were subtly encouraged not to select training clips, raise concerns or tick low challenge box.’ E50.

Groups too large with insufficient opportunity for interaction: ‘Smaller groups would be better with more chance to ask questions or discuss issues.’ E115. ‘Zoom meetings weren't that helpful and needed some better facilitation to get people to engage.’ E111

*Time for marking sufficient vs tight*

Examiners varied in whether they thought there was sufficient time for marking.

Sufficient time: ‘In general, I would say the day worked very well and there was sufficient time to mark all 26 candidates.’ E22

Tight timescales: Marking the cases was sometimes a bit tight; I had to skip mornings breaks to get ahead in order to complete the afternoon marking; so shorter briefing would help. E10

**Recommendations for cases, candidates, judgments and support**

Several recommendations were put forward to remedy any shortfalls seen in the assessment.

*Recommendations for case selection and candidate information*

Advice on case selection: ‘Some areas such as type of consultations submitted, and range of cases need refining.’ E170

Clearer information for candidates: **‘**The other issue is about cheating or ‘That is what we do in normal practice ‘- some candidates were looking at information while doing the consultation. If it is acceptable, then every candidate should be informed of that and there should be equality (the fairness we promote all the time).’ E145

*Recommendations for examiner judgments*

More information provided on cases: ‘I would have liked candidates to be able to submit the photos of lesions etc. to enable me to assess management better.’ E8. ‘It would be helpful to have more information in the workbook in some of the cases re PMH [past medical history] of patient.’ E55.

More calibration and benchmarking: ‘More calibration clips would have been really helpful. We did not find out how our own marks compared against the expert panel. More clips akin to the palette we saw (e.g. low challenge cases) would have helped us to feel more reliable and consistent across examiners in our judgements on the day.’ E42. ‘Needs more training audio recordings/videos with agreed scores and justifications as a way to set some sort of benchmark.’ E21

Clearer pass/fail descriptors: ‘The descriptors for the different domains would benefit from more clearly showing that the candidate needs to do enough to demonstrate competence i.e. that doing all that is needed in a low challenge case is not sufficient to gain a pass. The feedback statements need to reflect the above.’ E100 ‘Many consultations were a demonstration of consultation skills and not of clinical decision making. Often history taking was 'tell me more' and 'any other symptoms' and very formulaic with little evidence of focused history taking. Often there was a pre-existing diagnosis which the candidate simply confirmed. I think we are all agreed that we need new 'word pictures' to describe passing/failing.’ E86

Modify scoring to reflect consultation difficulty: ‘Change the generic descriptors to clarify that marks must be gained with the candidate starting with zero and gaining marks as they show skills. Telephone triage consults should score poorly because they can't lead to a definitive management plan. Giving the treatment the patient asked for should score zero in CM [Clinical Managament] if no additional value is added during the consultation. Candidates should not be rewarded for turning a 2-minute consult into 10.’ E108

Better feedback to examiners: ‘It would have been nice to get some feedback on the calibration exercise and some discussion on Zoom with fellow examiners would have been useful as part of the training.’ E33

Improved feedback to candidates: ‘We need some better feedback statements e.g. insufficient challenge to demonstrate skills, insufficient new management demonstrated. Some of the statements should also be in data collection e.g. insufficient psychosocial information to put problem in context.’ E102

Enhance training: ‘Training was good but the RCA needs to change really, and hence examiners will need further training.’ E177. ‘It would be good to have had examples of low challenge cases as these were the hardest to assess. It would be nice to know how other people marked the cases to give a bit of bench marking, and even some discussion of why they marked that way.’ E151

*Recommendations for training and support*

Alter balance of morning and afternoon cases: ‘Good to do a few afternoon cases in the morning.’ E76

Reduce meetings: ‘I have been very impressed by his well it has worked. I think now the meetings are becoming less useful as we become more familiar with the processes.’ E77

More one-to-one support: ‘Some of the issues raised would have been better dealt with on a one to one basis. ‘ E78
